# Pointing to One's Moving Hand: Putative Internal Models Do Not Contribute to Proprioceptive Acuity

**DOI:** 10.3389/fnhum.2018.00177

**Published:** 2018-05-15

**Authors:** Warren G. Darling, Brian M. Wall, Chris R. Coffman, Charles Capaday

**Affiliations:** ^1^Motor Control Laboratories, Department of Health and Human Physiology, University of Iowa, Iowa City, IA, United States; ^2^Bernstein Center for Computational Neuroscience, Bernstein Focus Neurotechnology Göttingen, Institute of Neurorehabilitation Engineering, Universitätsmedizin Göttingen, Georg-August-University, Göttingen, Germany

**Keywords:** kinesthesia, proprioception, hand, kinematics, internal model

## Abstract

We can easily and without sight bring our fingertip to our nose, or swat a mosquito on our arm. These actions rely on proprioception, also known as kinesthesia, which classically has been attributed to processing of sensory inflow by the CNS. However, internal model theories of sensorimotor neuroscience propose that proprioceptive localization also involves a contribution from estimates of limb kinematics derived from motor commands. We tested this prediction in 19 subjects who moved the right index finger tip to touch the moving left index finger tip under three conditions: (1) vision allowed, active movement of the left hand (2) vision blocked, active movement of the left hand, and (3) vision blocked, passive movement of the left hand imposed by the experimenter. The target left index finger tip was moved in a wide range of directions by unrestricted movements of the arm. Mean errors in apposition of the right to the left index finger tips were small, averaging <2 cm between sensors fixed to the finger nails. Note that the average distance between the sensors was ~1.7 cm when the fingertips were brought together in “perfect” apposition under visual guidance. The 3D mean distance and variable distance errors were marginally lower by some 2 mm with eyes open compared to the eyes closed active condition. However, mean distance and variable distance errors did not differ between the active and passive conditions with eyes closed. Thus, proprioceptive localization of one's moving hand is very accurate, essentially as accurate as when vision is allowed. More importantly, our results demonstrate that hypothesized internal model derived estimates of arm kinematics do not contribute to localization accuracy beyond that provided by sensory signals, casting doubt on their existence.

## Introduction

We know where, for example, our hands and digits are even if we are not looking at them and if we move them we have a sensation of their motion. This capacity, known as proprioception was called the “*muscular sense*” by Sherrington and is rightly referred to as the “*sixth sense*.” Classically, proprioception was thought to be subserved by sensory receptor signaling (e.g., see Matthews, 1981). However, it has been proposed that accurate motor performance depends on predictions of a CNS model to overcome noise in proprioceptive receptor signaling (Wolpert et al., 1995; Wolpert and Ghahramani, 2000). If so, the proprioceptive localization of a limb ought to be clearly much better when it is voluntarily moved, than if it were moved by an external agent. We previously investigated an example of such a task (Capaday et al., 2013), which involved asking subjects to apposition the index finger tip of one hand (reaching hand) to that of the other hand (target hand). The index finger tip of the target hand was localized with equal accuracy and with no greater variability when the target hand was moved actively by the subject, or passively by an experimenter. Moreover, we also observed in two subjects that localization accuracy was at best marginally better (by ~1 mm) when vision was allowed, suggesting that proprioception is remarkably accurate under the conditions experienced regularly in everyday life (i.e., unconstrained motion of the arm). We thus found no evidence for the operation of an internal model involved in proprioceptive localization as proposed by Wolpert et al. (1995).

Estimating kinematic variables, such as limb position and velocity, from operations of an internal model is an idea derived from modern control theory (e.g., Astrom and Murray, 2008). The process is referred to as state-estimation, of which the Kalman filter is an example. The idea is that, in principle, limb kinematic variables can be estimated from motor commands fed into a musculoskeletal forward model of the limb contained within the CNS. Furthermore, the predictions of the forward internal model are combined with the actual sensory inputs to obtain estimates of limb kinematic variables which ought to be more accurate and less variable than from either source alone. As mentioned, neither was found to be the case in our previous study (Capaday et al., 2013). However, the measurements of proprioceptive accuracy were made at the end of the target hand movements in all conditions, as was also the case in the Wolpert et al. (1995) study. Consequently, because at the end of a movement the state-estimation process may weight sensory inputs more than central estimates, it remains to be established whether proprioceptive localization is more accurate in the active vs. the passive condition while a movement is evolving and prediction of spatiotemporal aspects is needed to accurately localize the fingertip.

In the present study, therefore, our purpose was to determine proprioceptive accuracy when the target hand is in motion. The target hand being moved voluntarily by the subject, or passively by the experimenter. We tested the accuracy of index-to-index appositions for a variety of target hand motion directions and speeds. To be clear, we specifically tested the model proposed by Wolpert et al. (1995) which predicts better localization accuracy and lower variability in active vs. passive movement conditions (see also Wolpert and Kawato, 1998; Wolpert and Ghahramani, 2000). The model also predicts that subjects will undershoot the target in the passive condition, because the internal forward model is inoperative and normally provides a positive localization bias (i.e., overshoot). In the event, we found that proprioceptive localization of one's moving hand is equally accurate in active and passive conditions, with no difference in variability. Our results demonstrate that putative internal model derived estimates of arm kinematics do not contribute to localization accuracy beyond that provided by proprioceptive signals, casting doubt on their existence.

## Materials and methods

### Subjects

Nineteen subjects (10 males) age 18–22 years participated in this experiment. The study was approved by the local institutional review board and all subjects signed informed consent documents indicating their willingness to participate in the study.

### Task and conditions

Subjects began with the target (left) hand in front of the left shoulder with the index finger extended and pressing a switch. The subjects were instructed to move the target arm in a specified direction when the experimenter said “Go.” Movement of the target arm released the switch and a beep sound of 1 kHz, 200 ms duration, followed at random delays between 50 and 300 ms. The beep signaled the subject to begin moving the reaching (right) hand toward the target (left) hand to attempt to appose (i.e., touch) the respective index fingertips and hold them together for a second, or so. These delays were used for two reasons: (1) to prevent the subject from planning the left and right hand movements together to start simultaneously in the active target hand movement conditions, thereby requiring subjects to plan right hand movements on the basis of proprioceptive information from the target hand motion as in the passive target hand movement condition (see below for a complete description of the tasks) and (2) to allow for movements of different amplitudes and durations. We also used these random delays in the passive target hand movement condition to maintain constant instructions among the different conditions. Upon completion subjects returned the target hand to the switch. Subjects were instructed to make a single continuous movement toward the target hand fingertip while it was still moving. In summary, they had to intercept the target fingertip whilst it was in motion with the fingertip of the other hand.

Practice trials at the task were given before each condition to make sure that subjects understood the task, did not start reaching and target hand motion simultaneously, did not stop target hand motion before the reaching hand arrived, or bring the target hand toward the reaching hand. There were 3 experimental conditions: (1) eyes open, voluntary movement of target and reaching hands (VA), (2) no vision (blindfolded), voluntary movement of target and reaching hands (NVA), (3) no vision (blindfolded), experimenter passively moved the target hand along an approximately straight path in the desired direction, voluntary movement of reaching hand (NVP). The VA task was always done first and was followed by either the NVA or NVP task randomly. Importantly, subjects were not informed of the direction of the upcoming passive target hand motion in the NVP condition, but of course were instructed on the direction of target hand motion in the NVA condition. To examine whether there was a progressive task familiarity/practice effect during the VA task we assessed the relationship between errors and trial number. A significant negative correlation between error and trial number would indicate progressive improvement during performance of the VA task that might contribute to better performance when vision was not allowed due to practice of the task while could see their errors at the end of each trial. However, there were no significant negative correlations between error and trial number in the VA task for any subject. Thus, we are confident that performing the VA task first did not cause a progressive task familiarity/practice effect that inflated subsequent performance when only proprioceptive information was available.

**Figure 1:**
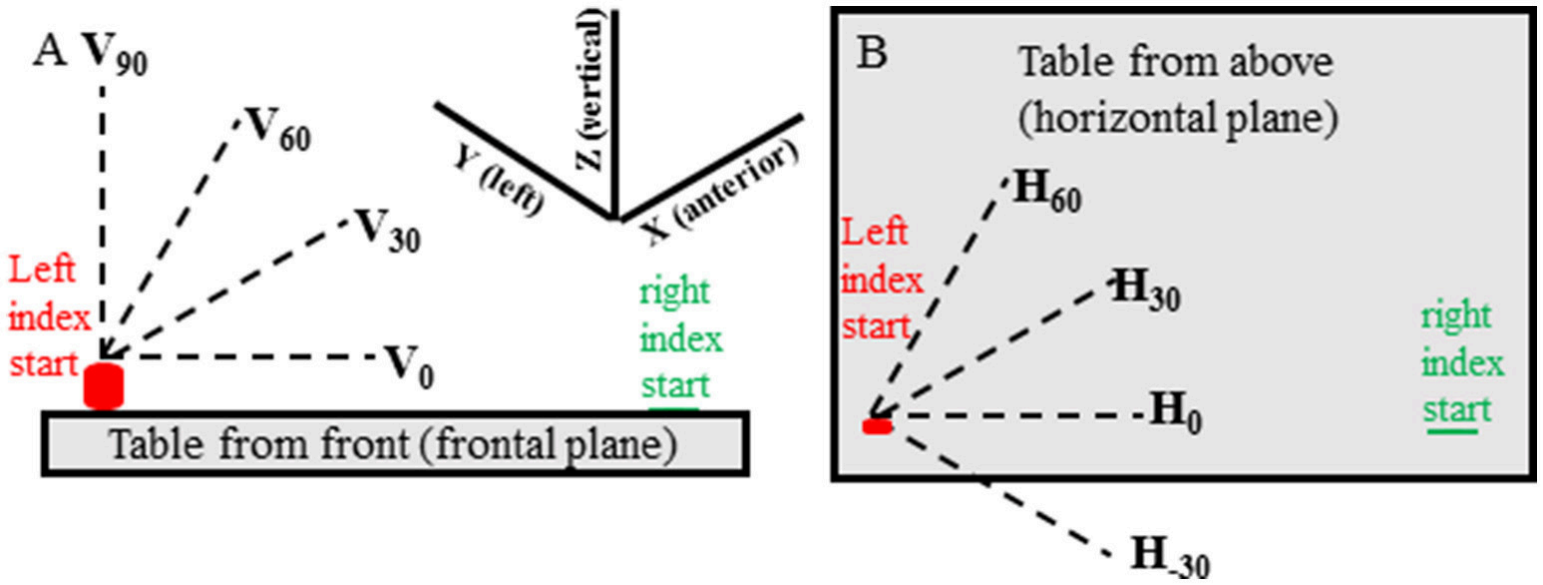
Target hand movement directions are shown relative to the table located in front of the subject and are shown from the subject's front view **(A)** and top view **(B)**. The target (left) hand started with the index tip on the cylinder containing a switch and was moved when the experimenter said “Go.” The pointing (right) hand started with the index tip on a small round mark on the table that could be felt by the subject. The pointing hand was moved when the subject heard a beep sound at random times, between 50 and 300 ms, after start of movement of the target hand.

In all experimental conditions movements of the target hand were made in 13 predefined directions in random order. There were 3 elevation directions (0°, 30°, 60° from horizontal—Figure 1A) for each of 4 horizontal (azimuth) directions (−30°, 0°, 30°, 60° from straight rightward—Figure 1B) and a vertical direction (90° elevation from horizontal) for a total of 13 directions (i.e., 4 horizontal × 3 elevation and 90° elevation). For each condition two movements were made in each direction for 26 movements and 78 movements in total for the 3 conditions. If target hand movement was not in the instructed direction, or if the target hand movement noticeably stopped before the reaching hand movement, the trial was discarded and repeated.

### Data acquisition

The positions of the left and right index fingertips were sampled at 240 Hz from a Trakstar system controlled by a custom written Matlab program. We used model-130 sensors (Ascension Technologies, Burlington, VT, USA) which were taped to the nails of the right and left index fingers. These sensors are very small measuring 1.5 × 7.7 mm. The sensor wires were taped to the hand and forearm with sufficient slack to allow natural unconstrained movements of both limbs. The Trakstar transmitter was located centrally on the table at a point beyond the motion workspace for each hand.

### Data reduction and analysis

**Figure 2:**
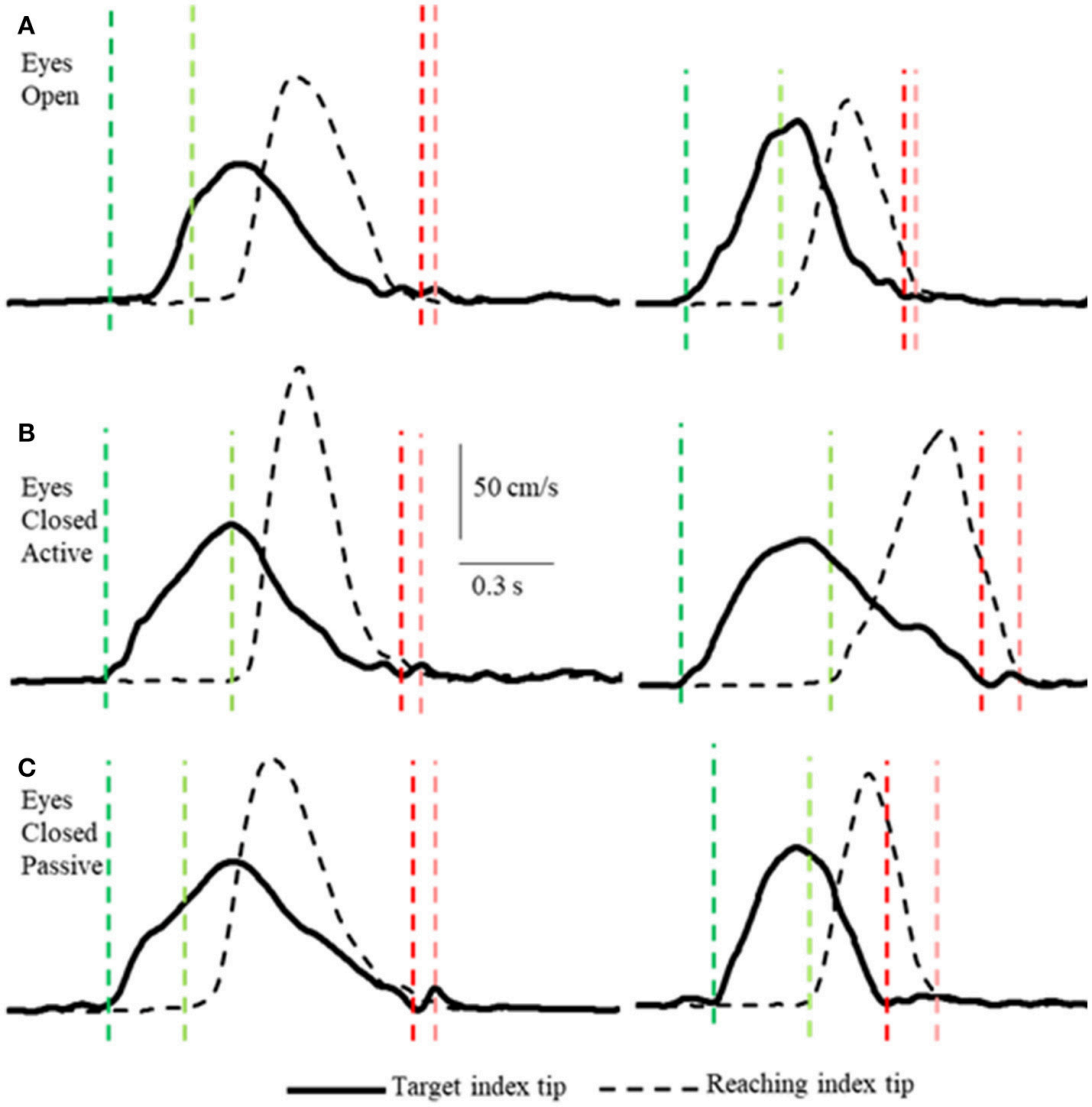
Examples of tangential velocity profiles of the index-tips of the target and reaching arms movements in the 3 conditions **(A–C)** by two subjects. The dashed vertical lines indicate onsets (greens) and terminations (reds) of motion of the target and reaching hands. Note that the velocity profiles of both hands are similar in all 3 conditions, including the passive condition when the target arm was moved by the experimenter. Also note that the time between onset of target and reaching hand movements varies and that both arms decelerate smoothly to movement terminations that are nearly simultaneous.

*The acquired* Matlab data files were imported into datapac2k2 (Run Technologies) for data analysis. The displacement data were filtered with a lowpass Butterworth digital filter (15 Hz rolloff frequency) and tangential speeds of each finger were computed and used to identify the onset and termination of movements of the two index fingers (e.g., Figure 2) using a velocity criterion of ~2 cm/s. The onset and termination times were subsequently verified by visual inspection of the records and any trial in which the subject did not move the reaching hand in a single continuous movement were eliminated from the analysis (average of < 1 in 78 movements eliminated per subject). The three-dimensional (3D) distance between right and left index fingertip sensors at the time of reaching fingertip movement termination were computed as a measure of apposition error. It is important to note for the correct interpretation of the measurements that will be presented that this distance cannot be zero, as the two sensors cannot be located at the same spatial coordinates. As a reference measure, therefore, consider that what may be termed “*perfect*” apposition of the finger tips (i.e., the subject deliberately placed the indexes tip-to-tip under visual guidance) produced inter-sensor distances averaging about 1.7 cm.

For each condition the mean 3D distance error and the variability of the distance errors (standard deviation) were calculated, as were the distance errors along each of the cardinal (X, Y, Z) directions (i.e., anterior/posterior, right/left, up/down). The instantaneous acceleration of the reaching and target index tips at reaching index tip movement termination was also measured to determine whether subjects voluntarily slowed the reaching finger as the target finger was approached, or whether subjects appeared to use contact with the target fingertip to stop the movement. Onset times of reaching fingertip motion relative to onset of target fingertip motion were also measured to determine whether the average and variability of these measures were similar across the three different experimental conditions.

Mean and variable 3D distance errors, as well as the distance errors in the cardinal directions were submitted to separate one-way repeated measures analysis of variance (ANOVA) to compare the localization accuracy among the three experimental conditions. Mean and variable errors in the 3 cardinal directions were also analyzed statistically using two-way (direction × condition) repeated measures ANOVAs. Huynh-Feldt adjustments to degrees of freedom were applied in all these ANOVAs if sphericity was violated and the corrected *p*-values are reported in the Results section. *Post-hoc* testing was completed using Tukey's HSD procedure. Effect sizes (Cohen's d) for the differences in mean and variable 3D distance errors between the NVA and NVP conditions were also computed and are reported in the Results. We also determined whether distance errors on individual trials in each subject varied predictably with the direction of target fingertip motion (azimuth and elevation movement directions computed assuming straight line motion of the target fingertip from movement onset to termination) using multiple linear regression.

We evaluated whether the characteristics of target and reaching hand movements and onset times for reaching hand movement relative to target hand movement were similar in the three experimental conditions. To this end we compared means of peak velocities of target hand movements and of times between onset of reaching hand motion relative to target hand motion onset. We also determined whether the reaching and target hand motions were voluntarily slowed prior to contact or whether contact of the target and reaching fingers was the primary cause of stopping their movements. This was done by measuring the deceleration durations and instantaneous tangential accelerations at the time of reaching hand movement termination (i.e., when tangential speed fell below 2 cm/s).

## Results

Four main sets of results are presented as follows. First we show that subjects intercepted the moving target fingertip accurately in all conditions, essentially as accurately in the NVA and NVP tasks as in the VA and that the variability of the distance errors were only marginally greater. Second, we report that the magnitude of the distance errors (i.e., measure of the apposition accuracy) were independent of movement duration. Lastly, we will consider any differences in the way movements were made in the different tasks.

Unlike, for example, clapping the hands together apposition of the two index finger tips while they are in motion is a task requiring precision, with or without vision. Subjects executed the task as follows. In the active conditions (VA and NVA) the reaching hand approached close to the target hand when the latter was slowing down and coming to a stop (Figures 2, **4**). Similarly, in the NVP condition, when only proprioceptive sensations were available, the reaching hand approached close to the passively moved target hand when the latter was coming to a stop. The manner in which the subjects executed the tasks was not instructed, it was the natural choice of all subjects. Note that the reaching hand began to decelerate well before contact in all conditions (Figure 2). It should be clear from the single peaked bell-shaped velocity profile of the reaching hand (Figure 2) that in all conditions the subjects estimated well the trajectory of the target hand from the outset and moved to it in a single movement.

**Figure 3:**
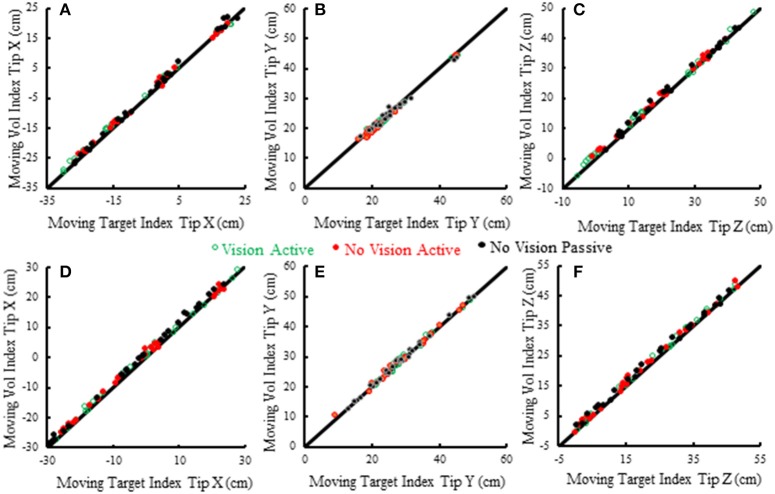
Scatterplots of voluntary (vol) right moving fingertip X-position (anterior-posterior), Y-position (right-left) and Z-position (vertical) vs. target (left) moving fingertip X, Y, Z at movement termination. The data from two subjects is shown (S5—**A–C**, S6—**D–F**). Each plotted point is data from a single trial in a single condition. The origin of each graph is the average starting position of the right index fingertip. The line of identity is drawn on each graph in black. Note that the data points in all conditions fall nearly exactly on the line of identity.

**Figure 4:**
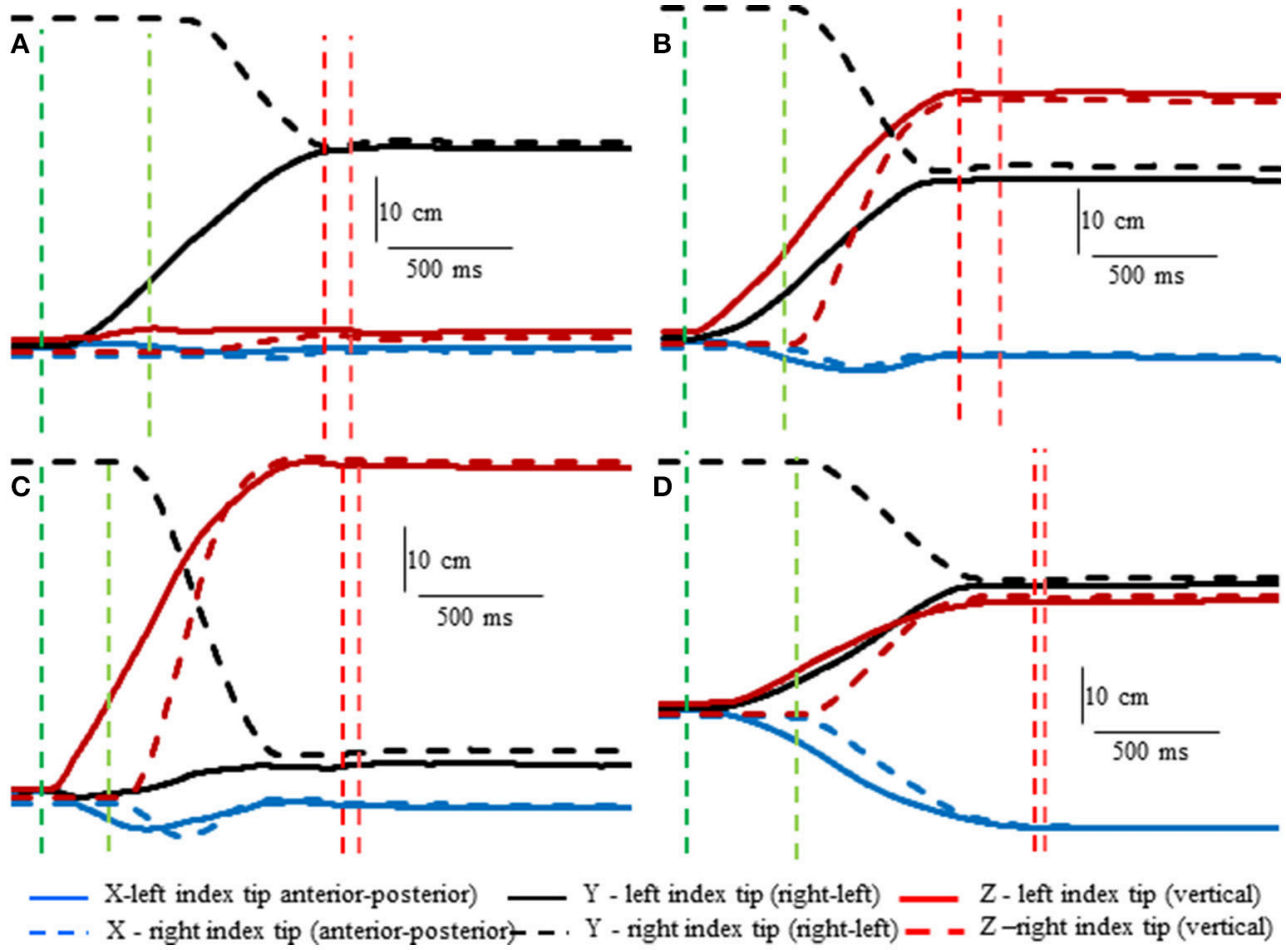
Examples showing right hand voluntary movements by a blindfolded subject to touch the right index tip to the left index tip when the left hand was moved by the experimenter (NVP condition). Position of the left index tip (target—moved by the experimenter) and right index tip (moved voluntarily by the subject—dashed lines) in 3D space are shown relative to the start position of the left index tip. The vertical lines indicate onset (greens) of left fingertip motion, onset of right fingertip motion, (reds) end of left fingertip motion and end of right fingertip motion. Note that the voluntarily moved right fingertip began motion at variable times after the left fingertip and that the two fingertips stopped moving at about the same time. **(A)** The left index was moved horizontally toward the right fingertip. **(B)** The left index was moved obliquely upward. **(C)** The left index was moved almost straight upward. **(D)** The left index was moved obliquely toward the subject.

**Figure 5:**
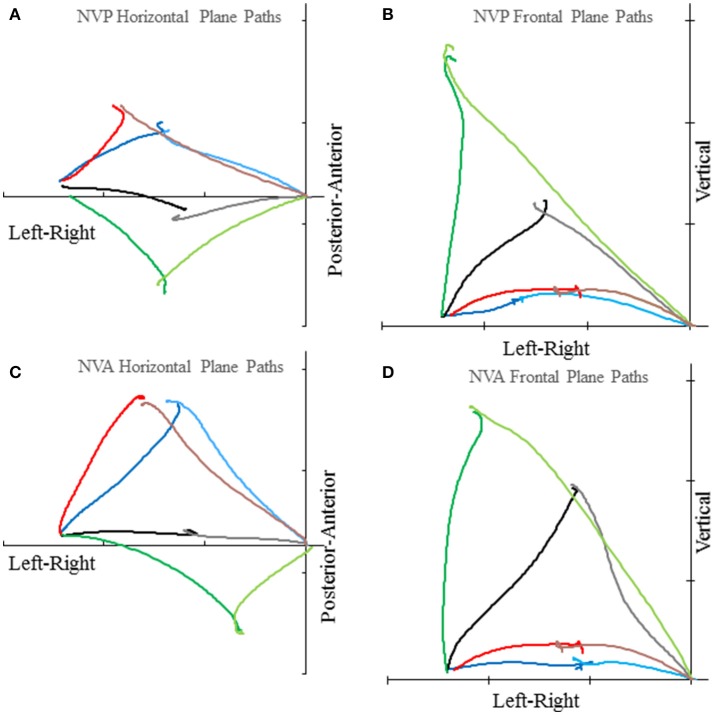
Examples of horizontal plane **(A**,**C)** and frontal plane **(B,D)** movement paths of the target and reaching index fingertips in the NVP and NVA conditions by subject S8. In each graph the plotted lines represent the reaching index fingertip voluntary movement (in the lighter color starting from near the origin) and target index fingertip passive movement (in the darker color starting about 51 cm left of the origin) for 4 trials with different directions (i.e., H30°V0°, H0°V0°, H30°V0°, H 60°V0° in the horizontal plane graphs and H0°V0°, H0°V30°, H0°V60°, H0°V90° in the frontal plane graphs). Tick marks on each axis represent 20 cm. Note the precise apposition of the two fingertips in all examples.

Subjects made remarkably accurate movements to the moving target fingertip over a wide range of distances and directions with, or without vision, and whether the movement of the target hand was active or passive. The accuracy of the proprioceptively guided movements in comparison to the visually guided movements is clearly observable in the scatterplots of Figure 3. The graphs show the endpoints in the three cardinal directions of the target and reaching fingertips under the different conditions for two subjects. Note that the data points fall nearly exactly on the line of identity in all conditions. Similarly, Figure 4 shows the displacement traces of the left and right index fingertips in the NVP task for movements in various directions. All movements were smooth and ended with the right index-tip usually touching the distal phalanx of the left index, with the two hands stopping nearly simultaneously (Figure 4). The accuracy of performance while blindfolded was also evident in horizontal and frontal plane plots of movement paths where it is clear that the reaching hand is directed in nearly the correct direction to intercept the target hand at the outset of its motion (Figure 5). This observation is important because it shows that purely proprioceptive information from the initial motion of the target limb in the passive condition—when the subject did not know the planned target movement direction before its motion—is used to appropriately direct the reaching limb to intercept the target limb.

**Figure 6:**
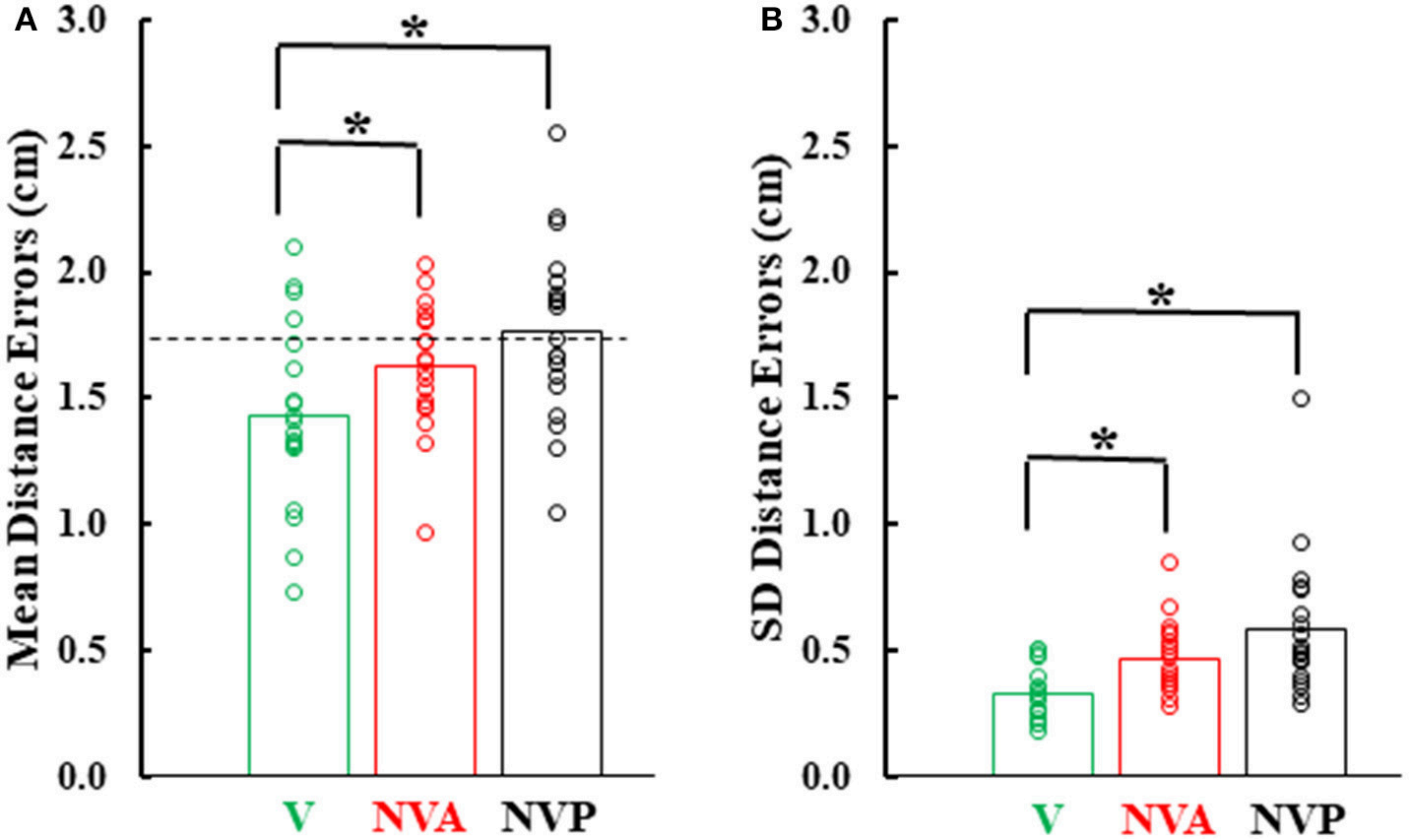
**(A)** Bar graphs showing mean distance errors and in **(B)**, mean distance variable errors across subjects in the 3 experimental conditions. The dashed line in **(A)** represents the mean distance error between the sensors when subjects slowly and deliberately touched their index fingertips. Also plotted on each bar are symbols showing the individual subject mean distance errors and distance variable errors. ^*^statistically significant (*p* < 0.001) differences between the vision (V) condition and the NV conditions (NVA, NVP).

Figure 6 provides a summary of the mean distance and variable errors in the three conditions studied. Mean distance errors were < 2 cm in all conditions for 15 of the 19 subjects and none of the subjects had mean distance errors exceeding 2.53 cm in any condition (Figure 6A). Because the distance between the fingertip sensors when subjects freely touched the index fingertips together under visual guidance averaged 1.7 cm (dashed horizontal line in Figure 6A), the actual mean distance errors were of the order of 1–2 mm, assuming the motion sensors could be co-localized. Variable distance errors were also quite low, averaging < 6 mm in each of the three conditions (Figure 6B). The small mean and variable distances errors, taken together, confirm the high accuracy in pointing to the index fingertip of the moving hand.

In comparing the mean and variable distance errors between experimental conditions small statistical differences were found. Statistical analysis revealed that mean distance errors differed among the three conditions [Figure 6A, *F*_(2, 36)_ = 15.85, *p* < 0.001]. *Post-hoc* testing showed that mean distance errors in the vision condition (1.44 cm) were very slightly lower (*p* < 0.001) than in the two no-vision conditions (1.63 cm in NVA and 1.76 cm in NVP) which had similar constant errors (*p* = 0.126, *d* = 0.58). Although there was a statistical difference between VA vs. NVA and NVP conditions, subjects were not necessarily more accurate in the VA condition because in so-called “perfect apposition” the mean distance error was 1.7 cm. Thus, in the VA task subjects simply brought their fingertips together slightly differently than in the other tasks, such as for example at a different angle between the fingers that put the index-tip sensors closer together than when subjects performed “perfect apposition.” The observation that the mean distance errors in the purely proprioceptive tasks are comparable to that of “perfect apposition” suggests vision made negligible contribution to the task and reemphasizes the high accuracy of movements made under proprioceptive guidance.

Variable distance errors also differed slightly among the experimental conditions [Figure 6B, *F*_(2, 36)_ = 10.11, *p* < 0.001]. Compared to the visual condition, variable distance errors averaged 1.4 mm higher when vision was blocked during active movement of the target hand (*p* = 0.008) and 2.6 mm higher when the hand was passively moved by the experimenter (*p* < 0.001). The slight difference in the eyes closed passive condition was due to one of the 19 subjects (Figure 6B). The variable distance errors were not, however, significantly different between the two no vision conditions (*p* = 0.509, *d* = 0.60). In summary, we found no difference in localization accuracy or variability between the NVA and NVP tasks and, as for mean errors, vision minimally improved precision of task performance.

**Figure 7:**
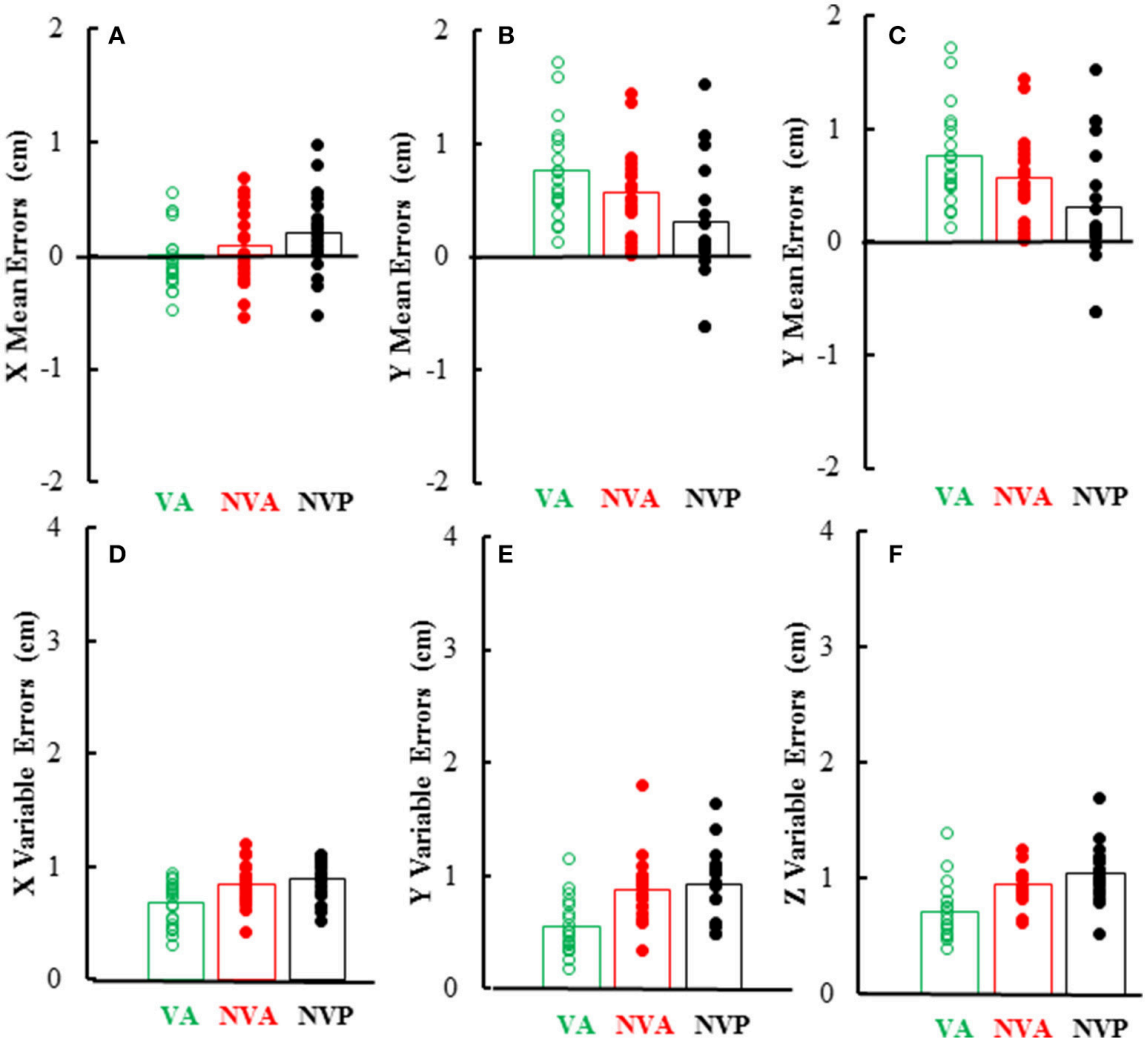
Bar graphs showing mean directional distance errors in each direction **(A–C)** and mean distance variable errors **(D–F)** in the 3 experimental conditions. Also plotted on each bar are symbols showing the individual subject mean directional distance errors **(A–C)** and distance variable errors **(D–F)**.

As can be inferred from the preceding, mean and variable errors in the 3 cardinal directions were minimally dependent on the movement conditions, however there were differences among directions. Mean errors averaged < 1 cm in each direction and were usually positive in the Y (left-right) direction (i.e., right index tip to right of left index tip) as would be expected, whereas errors in the other directions (X, Z) averaged near zero (Figures 7A–C). Statistical analysis of the magnitudes (absolute value) of mean errors showed that they were similar for the 3 conditions [*F*_(2, 36)_ = 0.36, *p* = 0.621], but differed among the 3 cardinal directions [*F*_(2, 36)_ = 9.38, *p* = 0.002], being higher in the Y (right-left) direction than in the other directions. Variable errors in the 3 cardinal directions also averaged < 1 cm (Figures 7D–F) and were slightly larger in the no vision conditions than in the vision condition [*F*_(2, 36)_ = 23.98, *p* < 0.001; *p* < 0.05 for *post-hoc* comparisons]. Variable errors also differed among the 3 directions [*F*_(2, 36)_ = 7.03, *p* = 0.003], being slightly higher in the vertical (Z) direction than in the other directions (X, Y).

**Figure 8:**
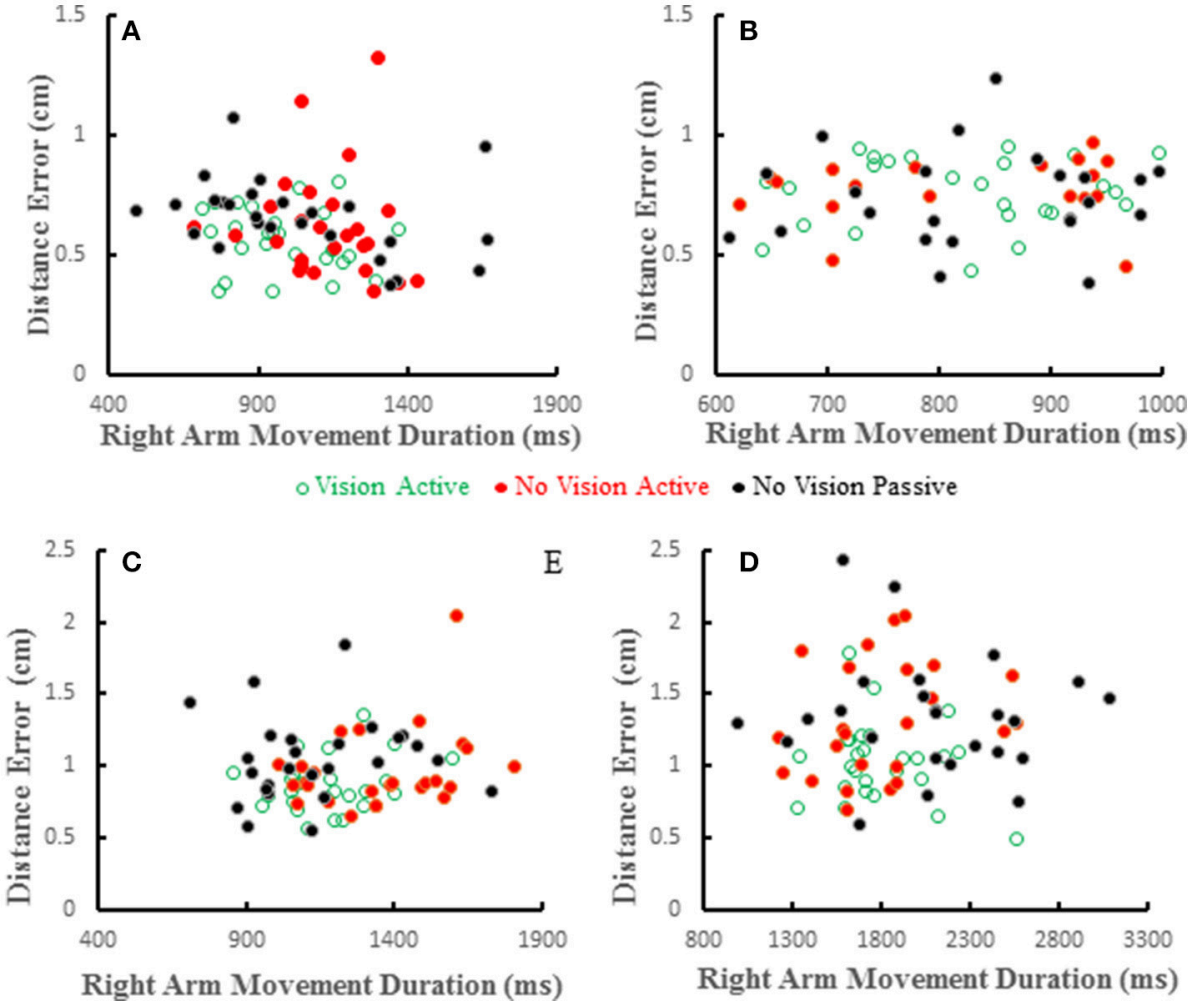
Scatter plots showing distance errors vs. pointing arm movement durations for individual trials in the 3 experimental conditions for 4 subjects **(A–D)**. Errors were distributed in a similar random manner in the 3 conditions and where uncorrelated with movement duration in any condition.

Distance errors on individual trials were poorly correlated with target hand movement directions. Coefficients of determination averaged < 0.2 across all subjects and conditions and were similar among the three conditions [*F*_(2, 36)_ = 0.226, *p* = 0.8]. This shows that distance errors were not dependent on target hand movement directions. We also studied the relation between distance error and reaching hand movement duration, which implies movement amplitude as the two are proportional. Wolpert et al. (1995) reported that under proprioceptive guidance subjects overestimated the location of their thumb. The bias errors increased monotonically peaking for movement durations of about 1 s and decreased for longer durations, but the bias errors were always positive (i.e., an overestimate). We did not replicate this observation in our previous study (Capaday et al., 2013). In both studies proprioceptive localization accuracy was measured at the end of the target hand movement. Here we have reexamined the issue whilst the target hand was in motion. Examples from four subjects are shown in Figure 8. Distance errors were independent of movement duration in all conditions. It is clear that there is no trend between these two variables, even when the data of all subjects are pooled together. More importantly, in the passive condition subjects did not undershoot the target as can be seen in Figures 6A, 8, contrary to the predictions of the hypothesis by Wolpert et al. (1995).

Lastly, we consider any potential differences in the characteristics of the movements made in the different conditions. Deceleration of the reaching hand was primarily voluntary rather than due to contact with the target finger in each condition (Table 1). This is clearly shown in the examples of Figure 2 as tangential velocity decreases smoothly to almost zero prior to contact of the reaching and target fingers. Deceleration durations averaged over 600 ms for the reaching hand and 875 ms for the target hand, showing that deceleration occurred over a prolonged period in both hands (Table 1). Instantaneous accelerations averaged < 70 cm/s at the termination of reaching hand motion showing that deceleration of both hands was largely completed before termination of reaching hand movement. These were similar across conditions although deceleration durations were shorter and instantaneous accelerations lower in the visual condition as would be expected. This demonstrates that subjects were actually reaching to the target index and not simply hitting it by chance. Overall, the results demonstrate that subjects used proprioceptive information on the initial motion of the target index fingertip to plan an appropriate movement to intercept it (Figures 4, 5).

**Table 1:** Averages of: deceleration durations and instantaneous tangential accelerations of the reaching and target index motion at the time of reaching hand movement termination (2 cm/s threshold), peak target and reaching hand speeds, and time between start of target and voluntary hand motions.

**Variable**	**VA**	**NVA**	**NVP**
Reaching index decel. dur. (ms)	875.7	952.3	986.7
Target index decel. dur. (ms)	609.7	696.2	646.7
Reaching index decel^a^ (cm/s/s)	−58.0	−69.3	−70.3
Target index decel^b^ (cm/s/s)	−56.9	−63.7	−65.4
Peak target index speed (cm/s)	66.61	61.07	57.59
Peak reaching index speed (cm/s)	104.4	98.3	101.94
Time between onsets of hand motions^c^ (ms)	341.3	382.2	373.2

a *Instantaneous acceleration of reaching index at reaching index movement termination*.

b *Instantaneous acceleration of target index at reaching index movement termination*.

c *Time between onset of target hand motion and reaching hand motion (note reaching hand motion onset was initiated after a sound occurring randomly between 50 and 300 ms after target hand motion onset)*.

The time between start of target and voluntary hand movements, deceleration of the voluntary hand prior to contact with the target hand and peak speed of the target hand motion differed somewhat among conditions. As expected, the start of reaching hand movement after target hand movement averaged about 10% shorter when vision was allowed than in the two no-vision conditions, indicating a longer time to start reaching hand movement after the sound following target hand motion onset when vision was blocked (Table 1). Peak speed of target hand motion was usually highest in the vision condition and lowest in the no-vision passive condition, as expected (Table 1). However, there were no differences in peak speed between the two no-vision conditions (*p* = 0.338), indicating that the experimenter moved the target hand at similar speeds to those during active target hand movement without vision. Peak speed of reaching hand motion was similar in all three conditions (Table 1). In summary, movement characteristics in the different tasks were in the main rather similar.

## Discussion

**Figure 9:**
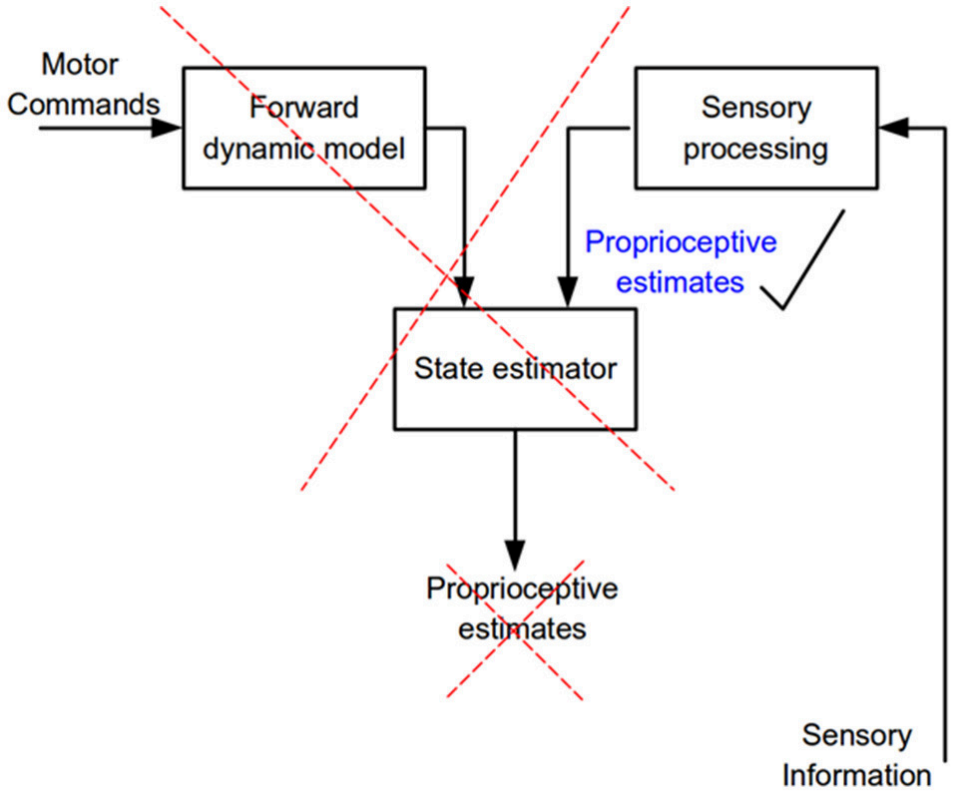
Flow of neural information involved in the estimation of limb kinematic variables. According to internal model theory a state estimator combines and weights processed sensory information and the output of a forward dynamics model to predict kinematic variables of a commanded movement (proprioceptive estimates). The present results show that only sensory information is needed to accurately estimate kinematic variables, no evidence for the operation of a state estimator was found.

The main question addressed in this study was whether proprioceptive acuity is better during active voluntary arm movements compared to imposed passive arm movements. The answer to this question provides direct evidence as to whether in the active condition an internal model based state-estimation process contributes to proprioceptive localization. There are two potential sources of information in the active condition, prediction from central estimates and sensory inputs due to target arm motion. In the passive condition only sensory information is available. No differences in either localization accuracy or variability were found between the two purely proprioceptive tasks. One may conclude that, either internal model based state estimates are no better than those derived from sensory information on its own, or that proprioception is not based on internal model operations. Following Occam's razor, the latter conclusion is the more sensible. Simply put, our results show that proprioception for control of upper limb movements is subserved by central processing of sensory inputs. The nature of this processing does not include internal model based state-estimation operations (Figure 9). Whether monitoring of motor commands and internal models might contribute to conscious perception of upper limb position and motion as suggested by some investigators (e.g., see Weeks et al., 2017) is not the focus of the present study. The reader is referred to the discussion in Capaday et al. (2013) for further details on this matter.

The NVA and NVP tasks we studied involved unconstrained movements in 3D using proprioceptive information as commonly occurs in everyday life. We can swat a mosquito on one arm with the hand of the other, tie knots, clap our hands, or reach to any part of our body, all without sight, because proprioceptive inputs provide accurate kinematic information throughout the movements. Requiring blindfolded subjects to touch the tip of the index finger whilst it is in motion is a test at the limits of proprioceptive acuity, necessitating accurate knowledge of the evolving arm configuration. Human subjects can perform this task very accurately as shown by the data presented in Figures 3, 6. Their accuracy at intercepting the moving target index finger tip with the reaching index fingertip is about as good as it is when slowly and deliberately touching the index fingertips under visual guidance. Furthermore, in the passive condition subjects did not know the direction of the imposed movement whereas, of course, in the active condition they did. Thus, unlike the study by Capaday et al. (2013) in which subjects had sensory information on target index fingertip position before starting the reaching hand movement, subjects in the present study had to estimate the target interception location from the initial (say over a few 100 ms) direction and speed of motion of the target index fingertip. A major reason for hypothesizing the existence of an internal model based state-estimation process is that sensory signals are noisy (e.g., Wolpert and Ghahramani, 2000). Clearly, in neither condition are sensory signals so noisy as to preclude accurate performance. Moreover, we observed similar and low variable errors in active and passive conditions with vision blocked, showing that there is no need for an internal model based state-estimation process to correct for noisy sensory signals as hypothesized. In fact, it is a basic principle of theoretical neuroscience that noise and ensemble averaging actually increase the information capacity of sensory receptors (e.g., see Stein, 1965; Moss et al., 2004; Hospedales et al., 2008).

A secondary purpose of our study was to compare movement guidance by proprioception vs. vision. To this end we compared localization accuracy when the finger apposition task was done with full visual and proprioceptive inputs available vs. when only proprioceptive sensory information could guide the task. When vision was allowed, localization accuracy was arguably minimally better than when only proprioceptive guidance of motion was allowed. What is important, is that our results clearly demonstrate that our proprioceptive sense provides highly accurate localization information, even when the targeted body part is in motion. And this implies that it also has accurate estimates of time varying variables, such as velocity and acceleration. To our knowledge, the present study is the first to provide a quantitative measure of the accuracy of proprioception during movement in comparison to that of vision. It should be made clear that what we have shown is that proprioceptive localization of a proprioceptively coded target is essentially as good as the visual localization of a visually coded target. Others have also noted the accuracy of proprioceptive guidance. For example, Soechting and Flanders (1989) showed that subjects make substantial errors, up to 15 cm, when pointing to remembered visual targets, with or without vision. By contrast, their accuracy was much better when reproducing their finger position after their arm had been moved by the experimenter.

Admittedly, proprioceptive inputs during active and passive movement are different. In the active condition γ-motoneurons, for example, are recruited and thus contribute to the discharge of muscle spindle afferents in active muscles (Vallbo, 1970, 1971). Presumably this does not occur in the passive condition. It remains an open issue how and if the CNS distinguishes γ-motoneuron mediated spindle afferent discharges from those due to muscle length changes alone (e.g., see Matthews, 1981). In any case, consider that in the active and passive condition spindle afferents in passively stretched muscles (“antagonists”) will signal length and velocity changes equally well. This sensory source has been shown in numerous studies to be used in the guidance of movements (e.g., see Capaday and Cooke, 1981, 1983; Cody et al., 1990; Inglis et al., 1991). This may be a main source of information used by subjects in proprioceptively guided motor tasks, such as in the present study.

Proprioceptive localization tasks at individual upper limb joints such as the shoulder and elbow when operating unsupported in 3D space are associated with mean absolute or rms errors in the range of 6–18° (Soechting and Ross, 1984; Darling, 1991; Darling and Miller, 1995; Hung and Darling, 2012). Such errors, if summed across these joints which produce most of the fingertip motion during voluntary arm movements, would suggest that we should be rather poor at localizing a fingertip. Indeed, a 5° error of estimate of shoulder orientation in one dimension (e.g., flexion/extension) could produce errors in fingertip location as large as 10 cm, but we have previously demonstrated much smaller errors in localizing the fingertip in 3D space (Capaday et al., 2013). Clearly, the CNS has access to more accurate sensory information concerning orientation of upper limb joints than can be inferred from single joint angle perception tasks. Because the upper limb is primarily used to position the hand and fingers to grasp external objects rather than to place the shoulder or elbow at specific joint angles, it seems likely that proprioception normally serves to specify arm endpoint (hand/fingertip) location, orientation and whole arm configuration during movements, rather than individual joint angles in perceptual task such as those commonly used to evaluate proprioceptive acuity. Indeed, the study of van Beers et al. (1998) showed that quantities related to joint angles are represented in the CNS much more accurately than they are consciously perceived, consistent with the remarkable accuracy of purely proprioceptively guided movements we have shown. Similarly, study of a backhand throwing elbow-wrist movement showed highly accurate proprioceptive temporal and spatial coordination of motion under active and passive elbow motion conditions (Cordo et al., 1995). These findings are consistent with the view that proprioception is normally used to automatically control movements, not to create conscious perceptions of limb orientation. To paraphrase Sherrington (1900), the “*muscular sense*” deals largely with the mutual relations between motile parts to guide movements.

The present results and those obtained when proprioceptive acuity was measured at movement termination (Capaday et al., 2013) are incompatible with the hypothesis that limb kinematic variables are derived from the operations of an internal model based state-estimation process (Figure 9). We have found neither increased accuracy nor decreased variance in the active condition and no bias in target localization, or change in bias between the active and passive conditions. Supporters of the state-estimation hypothesis may claim that the statistical analyses merely suggest that there is no reason to reject the null hypothesis, not that there is actually no difference between conditions. Note that the reason that statistical analysis did not detect a difference was not due to variability, as variability was the same in the NVA and NVP tasks. Furthermore, given the considerable number of subjects studied here and in our previous report (Capaday et al., 2013), even if there were a statistical difference—which statistical tests consistently fail to uncover—it is clear that it must be very small indeed. This raises the question as to the functional utility of such a hard to measure effect. Statistics is not a substitute for scientific reasoning and judgment and they cannot inform us on the scientific importance of a result. From close inspection of the data in Figures 3, 6 it is difficult to see what functional advantage is afforded by the active condition. Studies that report greater accuracy in active than in passive positioning tasks all rely on perceptual tasks that involve complex cortical processing (e.g., memory, transformation of proprioceptive coordinates into a visual frame of reference and conscious decisions about the equivalence of joint angles (see further details in Capaday et al., 2013). Yet, it is well-known that many organisms, including humans, perform difficult motor tasks accurately without vision and without conscious attention to details of motor performance. Proprioceptive acuity should be measured during such tasks and the mechanisms underlying control of such tasks should be the main focus of future investigations into the role of proprioception in the guidance of movements.

Our results and conclusion do not stand in isolation, other studies have reported findings inconsistent with the operation of internal model or corollary discharge based operations. For example, Monzee et al. (2003) showed that immediately following digital anesthesia the performance of subjects executing a well-learned object grasping and lifting task was severely disrupted. They commented that their findings were surprising in the light of numerous studies proposing that this task is driven by an internal inverse-model which ought to remain functional at least for a short time after sensory loss. They suggested that the putative internal model may require frequent sensory updating to maintain its function. An alternative explanation is that the task is not driven by an internal model. In a study of the timing of movement sensations evoked by transcranial magnetic stimulation of the motor cortex relative to direct electric stimulation of muscles, Ellaway et al. (2004) showed that the sense of movement depends on sensory feedback rather than on a central corollary discharge based mechanism. Relatedly, the sensation of the heaviness of objects has long been thought to be derived from an efference copy of motor commands (reviewed in, Proske and Gandevia, 2012). This prevailing view was contradicted by Luu et al. (2011) who showed cogently that under normal circumstances the sense of heaviness is due to sensory feedback, with a major component coming from muscle spindle afferents. Interestingly, two patients with a long standing large-fiber sensory neuropathy were also studied. When their thumb muscles were fatigued to half maximal force, the patients reported the lifted weight to be twice as heavy, by contrast to normal subjects who report a lighter weight (see details in Luu et al., 2011). At first sight the behavior of the patients seems as expected from the corollary discharge hypothesis. However, these patients cannot perform the task eyes closed, they rely on vision of the movements they initiate and base their judgment of heaviness on their onset, amplitude, and speed (Rothwell et al., 1982; Fleury et al., 1995). This study emphasizes, yet again, the crucial if not exclusive role of sensory feedback, here vision, in estimating the motor state.

We suggest that sensorimotor neuroscience should re-evaluate its position on internal model theories as a conceptual basis for the operation of the proprioceptive system. A number of cortical areas as well as the cerebellum are thought to subserve proprioception (e.g., Miall et al., 2007; Bhanpuri et al., 2013; Findlater et al., 2016). Attention should be concentrated on understanding how sensory inputs are processed within these structures to derive kinematic variables used in automatic posture and movement control. Our results help narrow the possibilities by pointing to the centripetal flow of sensory inputs and their processing within the CNS as the focus of research, rather than searching for purely central estimates of proprioceptive signals and mixtures of central and peripheral signals.

## Author contributions

WD: participated in experimental design, data collection, data analysis, manuscript preparation; BW and CRC: participated in data collection, data analysis, manuscript preparation; CC: participated in experimental design, data analysis, manuscript preparation.

### Conflict of interest statement

The authors declare that the research was conducted in the absence of any commercial or financial relationships that could be construed as a potential conflict of interest.
